# Dissipation of Triazole Residues and Their Impact on Quality Parameters and Nutrient Contents in Tomato Fruits and Products: From Farm to Table

**DOI:** 10.3390/toxics14010020

**Published:** 2025-12-24

**Authors:** Eman S. Elkholy, Atta A. Shalaby, Mahmoud M. Ramadan, Laila A. Al-Shuraym, Mustafa Shukry, Qichun Zhang, Ahmed A. A. Aioub, Rania M. Abd El-Hamid

**Affiliations:** 1 Plant Protection Department, Faculty of Agriculture, Zagazig University, Zagazig 44511, Egypta.aioub@zu.edu.eg (A.A.A.A.); 2 Department of Biology, College of Science, Princess Nourah Bint Abdulrahman University, P.O. Box 84428, Riyadh 11671, Saudi Arabia; 3 Department of Biomedical Sciences, College of Veterinary Medicine, King Faisal University, P.O. Box 400, Al-Ahsa 31982, Saudi Arabia; 4 State Key Laboratory of Soil Pollution Control and Safety, Key Laboratory of Environment Remediation and Ecological Health, Ministry of Education, Zhejiang University, Hangzhou 310058, China; 5 Agriculture Research Center, Central Agricultural Pesticide Laboratory, Pesticide Residues and Environmental Pollution Department, Dokki, Giza 12618, Egypt

**Keywords:** pesticide residue, degradation pattern, food safety, household processing, quality parameters, tomato fruits

## Abstract

Triazole fungicides are used to protect tomato yield from fungal infection. However, information regarding triazole residues and dissipation profiles is limited. This study aimed to evaluate the behavior, residue dissipation, and potential risks of penconazole (PCZ, 10% EC, 25 cm^3^/100 L water) and difenoconazole (DFZ, 25% EC, 50 cm^3^/100 L water) applied during the fruiting stage of tomatoes over 15 days in Mit Al-Qurashi village, Dakahlia Governorate, Egypt. The study also examined the residue levels of PCZ and DFZ in tomatoes following household preparation methods, as well as the health risks and residue intake associated with these pesticides. Additionally, the impact of PCZ and DFZ residues on macro- and micro-nutrient levels, as well as quality parameters in tomato fruits, was investigated. Our data showed that PCZ and DFZ exhibited dissipation rates recorded at 70.88% and 73.33% after 6 days of application, then increased to 99.74% and 98.25% after 15 days of application, respectively, corresponding to half-lives of 2.08 and 2.78 days. The pre-harvest intervals (PHIs) were determined to be 9 days for DFZ and 12 days for PCZ. Based on risk assessment and Health Risk Index (HRI) calculations, the withholding periods for using treated tomato fruits for human consumption were extended to 15 days for DFZ treatment and reduced to 9 days for PCZ. Notably, tomato fruits treated with PCZ or DFZ could be safely consumed one day after application if processed into paste. However, other forms of processing, including washing with water, acetic acid (5%), and sodium carbonate (5%) for 5 min, significantly reduced the residue levels of the tested fungicides. Moreover, the tested fungicides not only significantly reduced the levels of macro- and micronutrients in tomato fruits but also altered the quality parameters of the tomatoes. These findings could guide the safe and responsible use of PCZ and DFZ in tomatoes, helping to prevent potential health risks to consumers.

## 1. Introduction

Tomatoes (*Lycopersicon esculentum* Mill.) are among the most widely cultivated vegetables in Egypt [1]. The country produces approximately 7 million metric tons of tomatoes annually, with a cultivation area of around 221 thousand hectares. This accounts for roughly 34% of Egypt’s total vegetable cultivation area [2]. Tomatoes are mainly produced for local consumption as a nutritious source of vitamins and phytochemicals, including carotenoids, phenolic, and ascorbic acids [3]. Tomatoes are globally recognized as a fundamental staple, enjoyed daily in various forms, including raw, cooked, or processed into products such as canned goods, juice, or ketchup [4]. Tomatoes are vulnerable to various diseases and infestations throughout all stages of their development [5]. As a result, pesticides are used to protect against damage and help maintain both productivity and quality [6].

Triazole derivatives, including penconazole (PCZ) and difenoconazole (DFZ), are a key group of fungicides used to protect tomatoes from fungal infections [7] by inhibiting the demethylation process in fungi, specifically by blocking 14α-sterol-demethylase, which reduces the synthesis of ergosterol. This inhibition leads to the accumulation of 14α-methylsterols in the fungal plasma membrane, destabilizing the membrane and impairing enzyme function [8]. While the use of pesticides can effectively control plant diseases and boost yields, pesticide residues in food remain a concern [9] that can pose a potential threat to human health [10]. Therefore, to safeguard consumer health, it is essential to establish maximum residue limits (MRLs). The MRLs of PCZ and DFZ were 0.09 and 0.6 mg/kg, respectively [11,12].

Tomato processing involves several key steps, starting with harvesting from the field, followed by washing, refrigeration, peeling, blending, and heating to make paste (the final product) [13]. It has been observed that pesticide residues in agricultural products can be reduced during processing or through certain household preparation steps, such as washing, peeling, and cooking [14]. The impact of various culinary and processing methods, including washing, peeling, and cooking, on pesticide residues has been studied in a range of fruits and vegetables [15]. To our knowledge, there is no available information on the residual levels, degradation, and removal patterns of PCZ and DFZ in fresh or processed tomatoes. Our study aimed to determine the PCZ and DFZ degradation after one hour, 1, 3, 6, 9, 12, and 15 days of application in tomato fruits. Moreover, the dissipation of DFZ in tomato fruits under different processing conditions, including washing by water, acetic acid, sodium carbonate (Na_2_CO_3_), and heating to 100 °C to make tomato paste. Furthermore, the evaluation of chronic risks associated with long-term tomato consumption suggested a pre-harvest interval (PHI). The levels of macronutrients (nitrogen, phosphorus, potassium, and calcium), micronutrients (iron, manganese, and zinc), and quality parameters in tomato were also investigated under PCZ and DFZ treatments.

## 2. Materials and Methods

### 2.1. Fungicides and Reagents

The reference standards of penconazole (PCZ, >98.8% purity) and difenoconazole (DFZ, 99% purity) were obtained from Dr. Ehrenstorfer GmbH (Augsburg, Germany). The commercial formulation of PCZ (10% EC) and DFZ (25% EC) was obtained from the Central Agriculture Pesticides Laboratory in Giza, Egypt. Primary secondary amine (PSA, 40 µm Bondesil) and graphitized carbon black sorbent were obtained from Supelco (Bellefonte, PA, USA). Analytical-grade anhydrous magnesium sulfate (MgSO_4_), sodium chloride (NaCl), acetic acid, and sodium carbonate were provided by CARLO ERBA Reagents (Val de Reuil, France). HPLC-grade methanol, acetonitrile, and glacial acetic acid were obtained from Sigma GmbH (Darmstadt, Germany).

### 2.2. Field Experiment Setup and Procedures

The field experiment was carried out on tomato plants (*Solanum lycopersicum* L.) in a private field at Mit Al-Qurashi Village, Dakahlia governorate, Egypt (30.6717° N, 31.3652° E). The tomato plants were treated with the recommended dose of commercial pesticides PCZ and DFZ at 25 cm^3^/L and 50 cm^3^/L, respectively, by a motorized knapsack sprayer equipped with a single-nozzle boom according to the pest control program of the Ministry of Agriculture and Land Reclamation [16]. A control treatment was included, where no pesticides were applied. Each experimental plot was 30 m^2^ in size (15 × 2 m), with 50 cm between plants and 60 cm between rows to ensure optimal plant growth. The crop received the necessary irrigation, protection, and fertilizer treatments to guarantee an economically acceptable yield. Irrigation was provided using a drip irrigation system that delivered water directly to the root zone of each plant. During the trial, the highest recorded temperature was 28 °C and the lowest was 23 °C, while the average humidity ranged from 70 to 75%. No rainfall occurred during the trial period, and these weather conditions were typical for the region at the time of the study. Each treatment has three replicates. Tomato fruit samples (2 kg each) were randomly collected from designated plots at predefined intervals, including zero time (1 h), 1, 3, 6, 9, 12, and 15 days after treatment. The harvested samples were immediately placed in polyethylene bags and transported to the laboratory in an insulated ice container to preserve their integrity. Upon arrival, the fruits were coarsely chopped and homogenized using a HOBART food processor. The resulting homogenate was sealed in labeled polyethylene bags and stored at −20 °C until further processing and analysis.

### 2.3. Extraction and Purification of PCZ and DFZ Residues in Tomato Fruits

The residues of PCZ and DFZ were extracted using the QuEChERS (Quick, Easy, Cheap, Effective, Rugged, and Safe) method, as outlined by Lehotay [17]. Briefly, 10 g of tomato samples were placed into a 50 mL centrifuge tube, and 10 mL of acetonitrile containing 1% acetic acid was then added. The samples were shaken vigorously for one minute, after which 4 g of MgSO4 and 1 g of NaCl were added. Each tube was shaken immediately after the addition of the salt. The tubes were shaken vigorously for one minute, then centrifuged for five minutes at 4000 U min^−1^. A 1 mL aliquot of the supernatant was transferred to a dispersive clean-up tube containing 150 mg MgSO_4_, 10 mg GCB, 25 mg C18, and 25 mg PSA. The tubes were shaken for 30 s and then centrifuged for five minutes at 4000 U min^−1^.

### 2.4. HPLC Analysis

The cleaned extract was analyzed on HPLC (Agilent 1260 Infinity Series) (Agilent Technologies Deutschland GmbH, Böblingen, Germany), equipped with a quaternary pump and a diode array detector (DAD). The separation was achieved on a Nucleosil C18 analytical column (30 × 4.6 mm i.d., 5 µm particle size) with an autosampler injection valve. The mobile phase was eluted isocratically and consisted of acetonitrile and water in a ratio of 90:10 (*v*/*v*), delivered at a constant flow rate of 1.0 mL/min. The injection volume was set to 20 µL, with detection carried out at a wavelength of 205 nm. Under these conditions, the retention times were approximately 5.4 min for PCZ and 6.3 min for DFZ.

### 2.5. Method Validation

The performance of the HPLC method was assessed by evaluating several quality parameters, including linearity, accuracy, precision, matrix effect, recovery values, limits of quantification (LOQ), and limits of detection (LOD), in accordance with the EU SANTE/11312/2021 guideline [18]. The limits of quantification (LOQ) and detection (LOD) were determined using the equations outlined by Thomsen et al. [19]:

LOQ =10 S_0_/b, and LOD =3.3S_0_/b, (1)

where S_0_ is the calibration line standard deviation and b is the slope.

The linearity of the correlation coefficient (R^2^) was assessed using matrix-matched calibration curves and linear regression analysis by directly relating the area obtained from the measurements to the concentration of fungicides in the solvent. This analysis was performed at six distinct concentrations, ranging from 0.01 to 5 mg/L, as part of a six-point calibration curve. The matrix effect (ME) was evaluated by comparing the slopes of the standard curve using a solvent-based approach and the calibration curve using a matrix-matched approach [20].

Matrix effects (ME%) were determined using the following equation:

(2) $$ME\%=\frac{M\;matrix-M\;solvent}{M\;solvent}\;\times 100\%$$

where

ME is the matrix effect, and M matrix is the slope of the calibration curve in the matrix.

M solvent is the slope of the calibration curve in the pure solvent.

The recoveries of two tested fungicides at different fortification levels (0.1, 0.5, and 1.0 mg kg^−1^) were determined in five replicates using tomato samples to assess the accuracy and precision of the method.

### 2.6. Impact of Different Processing Conditions on Fungicide Residue Levels in Tomato Fruits

Residue removal experiments were conducted one day after fungicide application on tomato fruits, using different washing treatments or processing into tomato paste to reduce the levels of penconazole and difenoconazole. The collected fruit samples were divided into two main portions. The first portion was further subdivided into three replicates, each immersed for 5 min in a separate jar containing one of the following solutions: tap water, acetic acid (CH_3_COOH, 5%), and sodium carbonate (Na_2_CO_3_, 5%) [21]. After washing, the samples were air-dried on clean paper before packing. They were then crushed, extracted, and analyzed for residual fungicides.

The second portion was ground into small pieces using a Waring blender. The juice was then concentrated at 100 °C until a paste formed (30 min), with the addition of 2.5% NaCl [22]. The processed samples were then prepared for residue determination according to the previously outlined protocol.

Processing factors (PFs) were calculated for each transformation step according to Hakme et al. [23]

(3) $$\mathrm{PF}=\frac{Residues\;in\;processed\;products\;(mg/Kg)}{Residues\;in\;raw\;agricultural\;commodity\;(mg/Kg)}$$

A PF value less than 1 signifies a reduction in pesticide residues due to processing, whereas a PF greater than 1 reflects a concentration effect, indicating an increase in residue levels in the processed product, irrespective of weight or volume changes.

### 2.7. Risk Assessment

The estimated average daily intake (EADI, mg/kg bw) of CPZ and DFZ in tomatoes was calculated to assess the risk of long-term exposure. The Health Risk Indices (HRI) was then determined using the following formula, as described by Stephenson and Harris [24]

(4) $$\mathrm{EADI}=\mathrm{CRL}\;\times \;\frac{FI}{b.w}$$

(5) $$\mathrm{HRI}=\frac{EADI}{ADI}$$

where CRL is the residue concentrations of PCZ and DFZ in tomato, mg/kg; FI is the dietary consumption of tomato, (118 g/d) [25]; and b·w is the average body weight, assumed to be 80 kg [26].

Health Risk Indices (HRI), calculated based on the Good Agricultural Practices (GAP) guidelines, reflect the risk level associated with pesticide residues. The food is considered acceptable if the HRI value is less than one, which is equivalent to being below 100% of the Acceptable Daily Intake (ADI). If the HRI value exceeds 1 (greater than 100% of ADI), it is important to consider the potential risk to consumers and avoid consuming the food in concern [27].

### 2.8. Biochemical and Nutritional Indicators

To assess the impact of PCZ and DFZ residues on specific internal quality parameters and nutrients in both treated and untreated tomato fruits, samples were collected at 3, 6, and 9 days after application. The quality attributes analyzed included total soluble sugars, glucose, acidity, total soluble solids, ascorbic acid, β-carotene, protein, and dry matter content. Additionally, essential nutrients such as nitrogen (N), phosphorus (P), potassium (K), iron (Fe), manganese (Mn), calcium (Ca), and zinc (Zn) were quantified using previously established methods [28].

### 2.9. Kinetic Studies and Statistical Analysis

Statistical analysis was performed using one-way ANOVA, and mean values (mean ± SD) were compared using Tukey’s test in GraphPad Prism 10, with statistical significance set at *p* < 0.05. The degradation rate (k) and half-life value (t_1/2_) were determined using the equations described by Gomaa and Belal [29] as follows:

The degradation rate (k) = 2.303 × slope.(6)

Half-life value (t_1/2_) = 0.693 k^−1^. (7)

## 3. Results and Discussion

### 3.1. Analytical Method Validation for PCZ and DFZ Residues in Tomato Fruits

The evaluation of PCZ and DFZ in tomato fruits showed excellent linearity within the range of 0.01–5 mg/L, with high correlation coefficients (R^2^ = 0.9999 for PCZ and 0.9996 for DFZ). The slopes were 195.14 and 168.65, respectively, indicating strong calibration. Precision, measured as intra-day repeatability (RSDr), was ≤4.94% for PCZ and ≤3.22% for DFZ. The LOQ and LOD were 0.1 and 0.033 mg/kg for both fungicides, and matrix effects were 7.5% for PCZ and -15.3% for DFZ, respectively (Table 1). Our findings are consistent with those of Zhang et al. [30] and Abdallah et al. [31], who reported that the method validation for PCZ and DFZ closely aligns with our data, demonstrating efficiency, reliability, and excellent accuracy and repeatability. The recovery values for PCZ ranged from 98.44 to 105.08%, while those for DFZ were between 97.64 and 100.08%. The relative standard deviations (RSDs) ranged from 1.6 to 2.47% for PCZ and from 1.44 to 3.22% for DFZ (Table 2). These recovery values align with the acceptable range of 70–120% for pesticide residue analysis, as specified by international guidelines, including those from the U.S. Environmental Protection Agency (EPA) (OECD 2007). Likewise, the recovery values for PCZ and DFZ by the QuEChERS method ranged from 103.27 to 113.07% and 101.6 to 97% in tomato, respectively [4,32].

### 3.2. Persistence and Degradation of PCZ and DFZ Residues in Tomato Fruits

The persistence of PCZ and DFZ residues in tomato fruits was evaluated at intervals of 0, 1, 3, 6, 9, 12, and 15 days after treatment (Table 3). On day 0, the residues of PCZ and DFZ were 0.79 ± 0.12 mg/kg and 2.85 ± 0.24 mg/kg, respectively. After 3 days of treatment, the residue levels of PCZ and DFZ decreased to 0.44 ± 0.05 mg/kg (44.3% loss) and 0.91 ± 0.08 mg/kg (68.07% loss), respectively. By day 15, the residues of PCZ and DFZ were reduced to 0.002 ± 0.002 mg/kg and 0.05 ± 0.04 mg/kg, corresponding to degradation percentages of 99.74% and 98.25%, respectively. This variation is commonly observed and can be attributed to differences in the physicochemical properties of the fungicides. DFZ has a higher molecular weight, an octanol-water partition coefficient (Kow) equal to 4.36, and different water solubility compared to PCZ (Kow = 3.24), which can influence the initial retention and coverage on the fruit surface [33]. These properties can influence the initial retention, spreading, and penetration behavior on the fruit surface, leading to a higher initial loading of DFZ. DFZ exhibited rapid dissipation with a 68.07% loss within the first 3 days. This pattern is characteristic of the initial degradation phase, often driven by environmental processes such as photodegradation and washoff, and is coupled with rapid penetration into the plant cuticle [34]. Moreover, the dissipation of pesticides under real-field conditions is governed by a complex interaction of environmental factors, crop characteristics, pesticide properties, and application methods. Key factors include microclimatic conditions (such as temperature, humidity, air movement, and UV radiation), the growth stage at the time of application, plant species and morphology, as well as the chemical properties of the pesticide, including whether it is systemic or non-systemic. Collectively, these factors influence pesticide degradation processes, which are essential for modeling residue behavior and ensuring the safety of agricultural practices [35]. Abdallah et al. [36] reported that DFZ exhibits rapid initial dissipation in grapes, underscoring its consistent behavior across different matrices.

The maximum residue limits (MRL) for PCZ and DFZ were set at 0.09 mg/kg and 0.6 mg/kg, respectively, according to Codex standards. The pre-harvest intervals (PHI) were 12 days for PCZ and 9 days for DFZ. The respective half-life (t_1/2_) values were 2.08 days for PCZ and 2.78 days for DFZ, indicating the time it takes for half of the fungicide residues to degrade. The degradation constants (k) were 0.334 days ^−1^ for PCZ and 0.249 days ^−1^ for DFZ, representing the rate of fungicide breakdown in the treated tomato fruits. Our results align with those of Wu et al. [37] who found that the half-lives of DFZ ranged from 6.3 to 10.2 days in apples, 8.6 days in wheat plants [38], and 4.68 to 8.09 days in chili fruits [39]. Additionally, the t_1/2_ value of PCZ in soil is 89.1 days under field conditions and 117.2 days in the laboratory (at 20 °C), while the t_1/2_ value in crops varies from 1.5 to 14.0 days [33]. Another study showed that the t_1/2_ value of PCZ was found to be 125 h for unwashed tomatoes, respectively [40].

### 3.3. Processing Effects on PCZ and DFZ Residues in Tomato Fruits

The residues and degradation percentages of PCZ and DFZ in tomato fruits were evaluated across various processing conditions (unwashed, water-washed, acetic acid-washed, Na_2_CO_3_-washed, and paste) after one day of application (Table 4). The residues of PCZ and DFZ were 0.66 ± 0.14 mg/kg and 1.79 ± 0.27 mg/kg in the unwashed tomatoes, respectively. This aligns with previous research showing high residue concentrations shortly after pesticide application [41]. After washing with water, the residues of PCZ decreased to 0.44 ± 0.03 mg/kg (32.82% removal), while DFZ residues decreased to 0.31 ± 0.06 mg/kg (82.68% removal). This reduction is likely due to the removal of pesticide residues that are loosely bound to the surface of the fruit. Washing has been shown to reduce pesticide residues that are not strongly adhered, whereas peeling can eliminate those that have penetrated the fruit’s cuticle or skin [42]. Moreover, it has also been found that the effectiveness of washing in removing pesticide residues depends on the age of the chemical [43]. Our findings confirm the conclusions made by Romeh, Mekky, Ramadan and Hendawi [40], who reported that washing tomatoes reduced PCZ residue by 15% after one day of application. In addition, the washing process removed 89% and 86% of DFZ residue from Nandrin and Romane carrots, respectively [44]. Hendawi et al. [45] reported a 30% removal of imidacloprid when strawberries were washed for 3 min under running water. In the acetic acid-washed samples, PCZ and DFZ residues were further reduced to 0.38 ± 0.07 mg/kg (42.92% removal) and 0.41 ± 0.11 mg/kg (77.28% removal), respectively. This could be because acetic acid acts as a powerful chelating agent, rendering the residues less available compared to washing with water [46]. Our findings are backed by Randhawa et al. [47], who demonstrated that the highest reduction rates of imidacloprid were achieved with 9% acetic acid and citric acid treatments, resulting in reductions of 82.29% and 93.75% for cucumber, and 68.48% and 72.48% for pepper, respectively. In addition, the reduction in tebuconazole residue in grapes reached 74.45% with acetic acid [48]. Washing with sodium carbonate solutions is one of the preferred methods for eliminating pesticide residues from fruits and vegetables [49,50]. PCZ residues were undetectable, and DFZ residues were 0.30 ± 0.02 mg/kg, corresponding to 83.42% degradation in the Na_2_CO_3_-washed tomatoes. This can be attributed to the physicochemical properties of fungicides, as Na_2_CO_3_ has a notable degradation effect, especially on fungicide residues in citrus [51,52]. Furthermore, the degradation effect of Na_2_CO_3_ has been demonstrated for other types of pesticides and matrices [37]. Washing eggplant with 0.1% Na_2_CO_3_ resulted in a reduction of 75–87% in chlorpyrifos, fenitrothion, and malathion residues. Similarly, washing tomatoes with 5% Na_2_CO_3_ led to a 54% reduction in methomyl and a 69% reduction in acetamiprid residues [53]. Along the same lines, the tomato treated with water washing, sodium bicarbonate (5%), and acetic acid (5%) reduced the DFZ residue to 106, 74, and 68 mg kg^−1^ compared with the control (127 mg kg^−1^). In paste processing, PCZ residues were fully eliminated (100%), whereas DFZ residues were reduced by 94.97%. The high reduction values of the tested fungicides in the paste could be explained by the following factors: first, the heat pretreatment of the tomato fruits, which involved immersing the tomatoes in hot water at 100 °C for 30 min before peeling and processing; second, the removal of the fruit skins, where pesticide residues may have concentrated during straining; and third, the post-heat preservation treatment applied to the resulting pasteurized juice [54]. Also, the residue levels in tomato juice are influenced by the partitioning properties of the pesticide between the fruit skin/pulp and the juice. The pulp, which often contains the skin, retains a significant amount of lipophilic residues. As a result, moderately to highly lipophilic pesticides are poorly transferred into the juice, and their residues are further reduced during clarification processes such as centrifugation or filtration [55]. Processing factors (PF) are crucial in risk assessment, as they help refine exposure evaluations for consumers concerning pesticide residues in processed foods [56]. The processing factor for PCZ was 0.67, while DFZ showed a lower processing factor of 0.17. Acetic acid washing resulted in a processing factor of 0.58 for PCZ and 0.23 for DFZ, indicating a moderate reduction in fungicide residues. Washing with Na_2_CO_3_ resulted in a processing factor of 0 and 0.17 for both PCZ and DFZ. No processing factor was determined for the tomato paste for both fungicides. Chen et al. [57] reported that the PF of dinotefuran was 0.081 across the entire tomato paste processing, indicating that the processing reduced dinotefuran levels.

### 3.4. Changes in Health Risk and Residue Intake of PCZ and DFZ in Tomatoes Post-Application

Estimated Average daily intake (EADI), HRI values, and health risk assessment for PCZ and DFZ residues in tomato fruits during the experimental period are presented in Table 5. The health risk analysis was performed following the guidelines established by LeJeune et al. [58], which specifies that the estimated daily intake of pesticide residues should remain below the ADI to ensure consumer safety. The range of EADI values for PCZ was from 0.1156 to 0.0002 mg/kg, and for DFZ, it was from 0.4203 to 0.0073 mg/kg, spanning from the initial application to 15 days after application. Meanwhile, a gradual decrease in the HRI values for both PCZ and DFZ was observed from day zero to the end of the experiment. The HRI values were 3.88 for PCZ and 42.03 for DFZ initially, then decreased to 1.308 and 11.21, respectively, after 3 days of application. By day 15, the HRI values dropped further to 0.0098 for PCZ and 0.7375 for DFZ. The health risk assessment indicated that the initial risk from PCZ and DFZ residues was significant, with a “Yes” recorded for health risk from days 0 to 6 for PCZ. However, by day 15, the health risk for PCZ was rated “No,” and similarly, the health risk from DFZ diminished over time, reaching a “No” status by day 15. Our findings are supported by Malhat and Anagnostopoulos [59], who revealed that exceptionally low EADI values for etoxazole indicate minimal dietary exposure. The HRI values, ranging from 0.0001% to 0.007%, indicate that the use of etoxazole on okra presents a negligible risk to human health, even when considering the potential for higher application frequencies or dosages. Notably, these HRI values are well below the threshold of 1, further confirming the safety of the recommended application practices. Additionally, a recent study assessed the dietary risks of 57 active ingredients in Polish apples over a 17-year period. The highest acute risk identified was for tebuconazole, with a maximum concentration of 0.5 mg/kg, reaching 140.1% of the Acute Reference Dose (ARfD) for toddlers and 36.5% of the ARfD for adults [60]. The acceptable daily intake (ADI) for DFZ is 0.01 mg/kg body weight/day, and its residue levels on harvested crops range from 0.003 to 0.038 mg/kg. These residue levels are well below the ADI, indicating safe consumption. The percentage of ADI consumed varies by age: for children, it ranges from 0.72 to 9.10%, for adolescents from 0.32 to 4.11%, and for adults from 0.17 to 2.11% [61].

### 3.5. Evaluation of Fungicide Residues on Nutrients in Tomato Fruits

**Figure 1:**
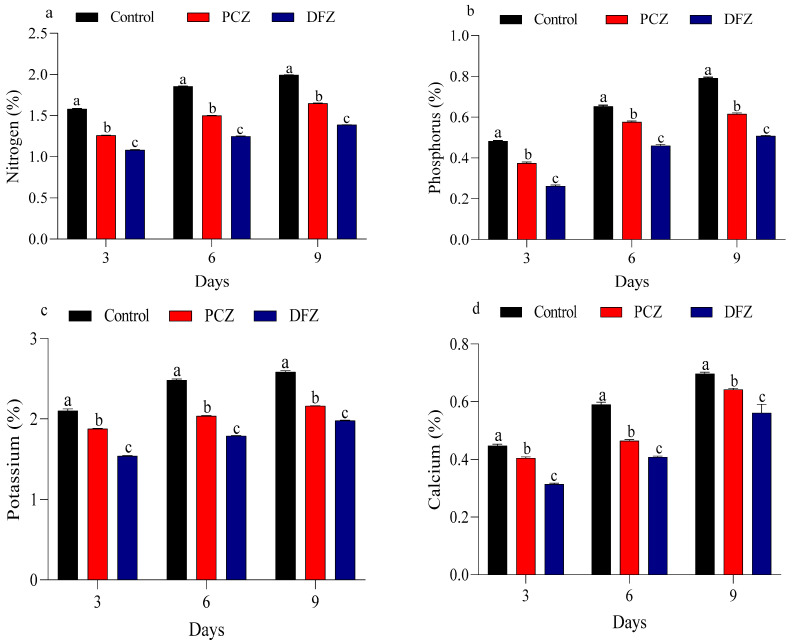
Impact of penconazole (PCZ) and difenoconazole (DFZ) on macronutrient levels in tomato fruits at 3, 6, and 9 days post-treatment: (**a**) Nitrogen, (**b**) Phosphorus, (**c**) Potassium, and (**d**) Calcium. Each column represents the mean ± SEM of three independent experiments. Different letters on top of the bar indicate significant differences (*p* < 0.05).

Our data showed that PCZ and DFZ have a significant effect (*p* < 0.05) on the macronutrient composition in tomato fruits, with a more pronounced reduction observed over time. The levels of macronutrients in tomato fruits subjected to PCZ and DFZ stress were evaluated at 3, 6, and 9 days after treatment (Figure 1). At 3 days post-application, both PCZ and DFZ treatments led to reductions in nitrogen, phosphorus, potassium, and calcium levels by 1.25, 0.37, 1.87, and 0.40%, respectively, compared to the control (1.58, 0.48, 2.10, and 0.44%). By 9 days post-application, the PCZ and DFZ treatments further decreased nitrogen levels from 1.99% to 1.64 and 1.38%, phosphorus levels from 0.79% to 0.61 and 0.50%, potassium levels from 2.58% to 2.16 and 1.97%, and calcium levels from 0.69% to 0.64 and 0.56%, respectively.

**Figure 2:**
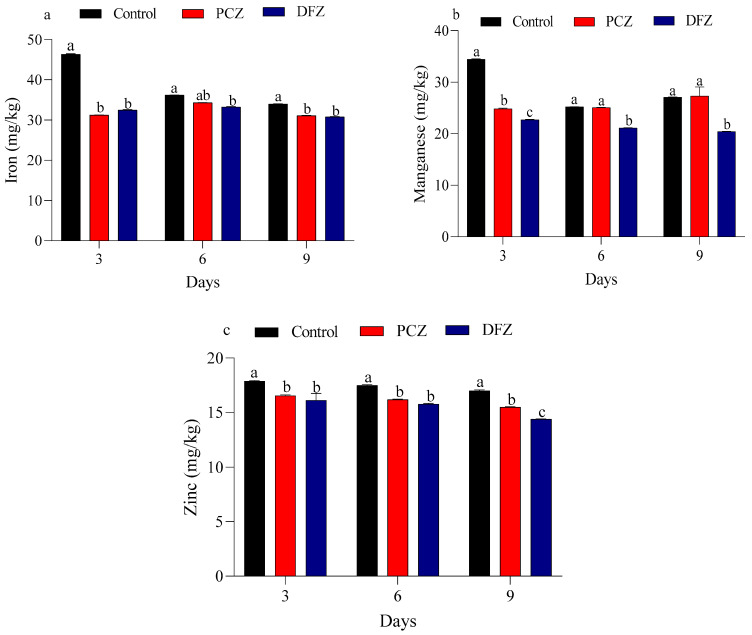
The micronutrient levels ((**a**) iron, (**b**) manganese, and (**c**) zinc) under penconazole (PCZ) and difenoconazole (DFZ) stress in tomato fruits at 3, 6, and 9 days post-treatment. Each column represents the mean ± SEM of three independent experiments. Different letters on top of the bar indicate significant differences (*p* < 0.05).

Figure 2 presents the levels of iron, manganese, and zinc measured at 3, 6, and 9 days after the application of PCZ and DFZ treatments, compared to the control group. The control group exhibits a gradual decrease in iron and magnesium levels from 46.320 and 34.46 mg/kg at day 3 to 33.987 and 27.09 mg/kg at day 9, indicating a consistent decline over time, respectively. Meanwhile, the control group shows a modest decline in Zn level from 17.877 mg/kg at day 3 to 16.997 mg/kg at day 9. The PCZ and DFZ treatments showed a slight fluctuation in iron levels with a small decrease at day 3 (31.227 and 32.48 mg/kg), followed by an increase at day 6 (34.30 and 33.24 mg/kg), and then a slight reduction again at day 9 (31.11 and 30.78 mg/kg). Manganese levels in tomato fruits show a modest increase under PCZ stress, rising from 24.84 mg/kg on day 3 to 27.30 mg/kg on day 9. Conversely, under DFZ stress, manganese levels decrease slightly, from 22.70 mg/kg on day 3 to 20.42 mg/kg on day 9. Tomato fruits subjected to PCZ and DFZ treatment demonstrate a slight reduction in zinc levels, rising from 16.54 and 16.12 mg/kg at day 3 to 15.48 and 14.39 mg/kg at day 9, respectively. The reasons for our result may be due to pesticide residues disrupting plant metabolism, interfering with nutrient uptake, and inducing stress responses [62]. Moreover, pesticides may inhibit the plant’s ability to absorb essential nutrients, damage photosynthetic processes, and alter the microbial communities in the soil, which are crucial for nutrient cycling [63]. In addition, pesticides can affect plant growth hormones, reducing overall plant growth and nutrient synthesis [64]. These combined factors lead to a decrease in macronutrients like nitrogen, phosphorus, potassium, and calcium in tomatoes. Our findings support the conclusions drawn by Shalaby [65], who reported that lambda-cyhalothrin residues significantly reduced the levels of all nutrients under study, including N%, P%, K%, iron (mg/kg), manganese (mg/kg), calcium (%), and zinc (%), in treated pepper fruits compared to untreated ones. Another study demonstrated that profenofos significantly lowered the mean levels of N, P, K, Ca, Fe, and Mn in tomato fruits compared to the untreated ones [28].

### 3.6. Changes in Tomato Fruit Quality Under PCZ and DFZ Stress

**Figure 3:**
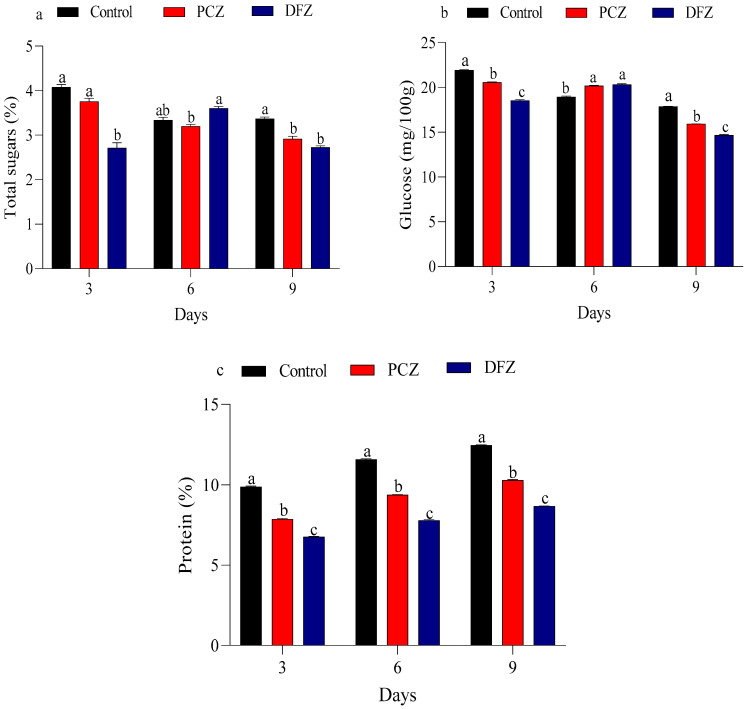
Effect of penconazole (PCZ) and difenoconazole (DFZ) on total sugar (**a**), glucose (**b**), and protein (**c**) in tomato fruits at 3, 6, and 9 days post-treatment. Each column represents the mean ± SEM of three independent experiments. Different letters on top of the bar indicate significant differences (*p* < 0.05).

Our results of total sugars, glucose, and protein content in tomato fruits under two tested fungicide stress at 3, 6, and 9 days post-treatment are presented in Figure 3. For total sugars, the control group showed a gradual decline, from 4.080% at day 3 to 3.370% at day 9. PCZ treatment led to a consistent reduction, with values starting at 3.756% at day 3, decreasing to 2.9167% by day 9. The DFZ treatment displayed a more variable response, beginning at 2.7160% at day 3, rising slightly to 3.011% at day 6, and then returning to 2.7267% by day 9 (Figure 3a). The control group exhibited a reduction in glucose content, from 21.91% at day 3 to 17.86% at day 9. Similarly, PCZ treatment resulted in a decrease, from 20.56% at day 3 to 15.91% by day 9. In contrast, DFZ treatment showed a reduction from 18.54% at day 3 to 14.67% at day 9, with a slightly less pronounced decline compared to the control and PCZ treatments (Figure 3b). On the other hand, the control group protein content exhibited a steady increase from 9.87% at day 3 to 12.45% at day 9. PCZ treatment caused a decline in protein content, starting at 7.86% at day 3 and rising slightly to 10.30% by day 9. Similarly, DFZ treatment increased from 6.76% at day 3 to 8.67% at day 9 (Figure 3c).

**Figure 4:**
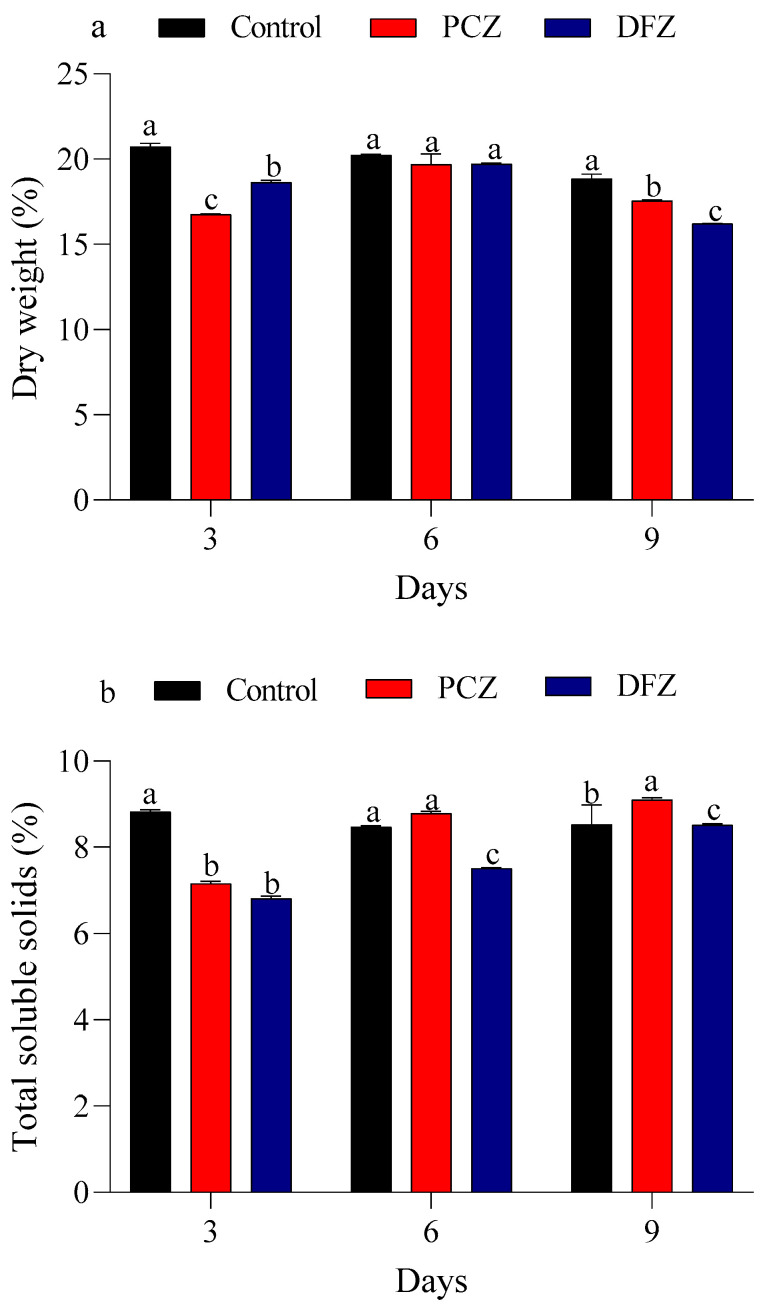
Changes in dry weight (**a**) and total soluble solids content (**b**) in tomato fruits following treatment with penconazole (PCZ) and difenoconazole (DFZ) at 3, 6, and 9 days post-treatment. Each column represents the mean ± SEM of three independent experiments. Different letters on top of the bar indicate significant differences (*p* < 0.05).

The effects of PCZ and DFZ treatments on dry weight and total soluble solids content in tomato fruits were assessed at 3-, 6-, and 9-day post-treatment (Figure 4). The dry weight of tomato fruits in the control group gradually decreased from 20.73% at day 3 to 18.85% at day 9. The PCZ and DFZ treatments showed a fluctuation in dry weight, starting at 16.7 and 18.64% at day 3, increasing to 19.69 and 19.71% at day 6, and then slightly decreasing to 17.54 and 16.21% at day 9, respectively (Figure 4a). For total soluble solids, the control group showed a slight decrease from 8.82% at day 3 to 8.46% at day 6, with a small recovery at day 9 (8.52%). The PCZ and DFZ treatments exhibited an increase in total soluble solids from 7.16 and 6.81% at day 3 to 9.10 and 8.51% at day 9, respectively (Figure 4b).

**Figure 5:**
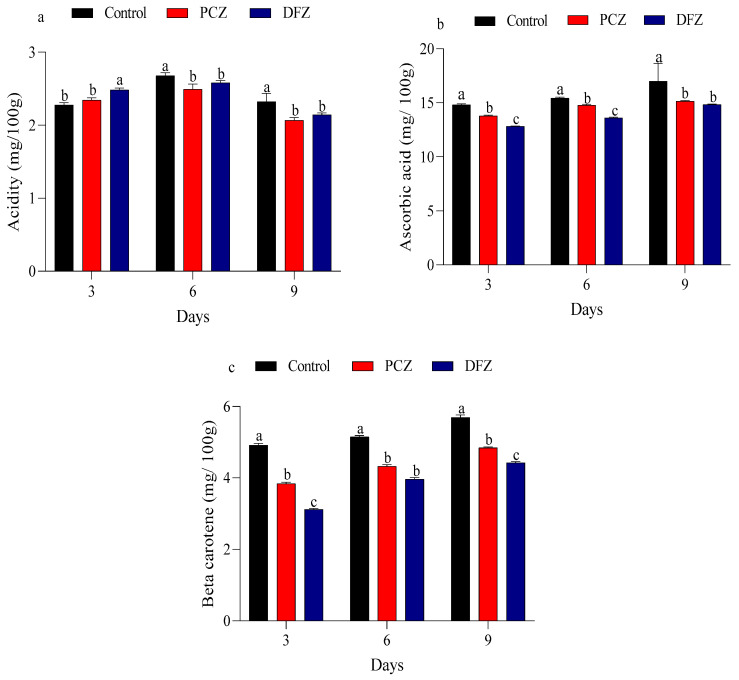
Variation in acidity (**a**), ascorbic acid content (**b**), and beta carotene content (**c**) of tomato fruits following treatment with penconazole (PCZ) and difenoconazole (DFZ) at 3, 6, and 9 days after treatment. Each column represents the mean ± SEM of three independent experiments. Different letters on top of the bar indicate significant differences (*p* < 0.05).

Changes in acidity, ascorbic acid, and beta carotene content of tomato fruits were assessed following treatment with PCZ and DFZ at 3-, 6-, and 9-day post-application (Figure 5). In the control group, acidity increased from 2.27 mg/100 g at day 3 to 2.68 mg/100 g at day 6, followed by a slight decrease to 2.32 mg/100 g at day 9. In contrast, the PCZ and DFZ-treated group exhibited a reduction in acidity from 2.03 and 2.84 mg/100 g at day 3 to 2.49 and 2.52 mg/100 g at day 6, and a further decline to 2.06 and 2.14 mg/100 g at day 9 (Figure 5a). The ascorbic acid content in the PCZ and DFZ treatments was consistently lower compared to the control group where the ascorbic acid content in the control, PCZ, and DFZ treatments demonstrated a gradual increase, rising from 14.82, 13.79, and 12.82 mg/100 g at day 3 to 15.43, 14.78, and 13.60 mg/100 g at day 6, and reaching 17.00, 15.15, and 14.83 mg/100 g at day 9 (Figure 5b). On the other hand, PCZ and DFZ treatments significantly decrease (*p* < 0.05) the beta carotene levels compared with the control. However, the control, PCZ, and DFZ groups increased progressively from 4.91, 3.84, and 3.11 mg/100 g at day 3 to 5.69, 4.84, and 4.42 mg/100 g at day 9 (Figure 5c).

The reasons behind the changes in the quality parameters by PCZ and DFZ may be due to both fungicides affecting their biochemical reactions and functions. Moreover, plants were affected by pesticides in several ways; these include inhibition of photosynthesis, mitosis, and cell division [66]. In addition, pesticides can interfere with the activities/functions of plant enzymes, root growth, synthesis of chlorophylls and proteins, and the destruction of cell membranes [66]. Notably, Taiz and Zeiger [67] found that the higher concentrations of pesticides could retard the physiological and biochemical processes in plants, which could provide further insights into the retardation in growth. In addition, these differences may be attributed to altered nutrient transport from the soil, as a portion of foliar-applied triazoles can reach the soil [68]. Triazoles are known to form complexes with divalent cations, which can lead to unexpected changes in their chemical behavior [69]. Additionally, the presence of triazoles may affect enzymes or physiological mechanisms involved in the regulation of macro- and micronutrient uptake and distribution, as well as influence enzyme levels and even gene expression [70]. Interestingly, triazole acts as a plant growth regulator and also influences hormonal balance, photosynthetic rate, enzyme activities, lipid peroxidation, and yield components in various crop plants [71]. Triazole inhibits the cytochrome P-450-mediated oxidative demethylation reaction, as well as those that are necessary for the synthesis of ergosterol and the conversion of kaurene to kaurenoic acid in a gibberellin biosynthesis pathway [72]. Our results align with Hýsková et al. [73], who reported that PCZ can protect the tomato yield against fungal infection but also influence the quality of the tomato fruit’s nutrition as a side effect. Another study demonstrated that the significant differences in tomato quality parameters may relate to abiotic stress caused by PCZ. Also, triazole-induced inhibition of gibberellin biosynthesis and increased cytokinin content may enhance root growth [74] and elevate sugar levels in the leaves and tubers of cassava (*Manihot esculenta* Crantz) [75]. Kovač et al. [76] found PCZ forms stable complexes with Zn by often losing a proton (deprotonate) to bond with Zn, creating stable complexes like [Zn(PCZ-H)^+^ or [Zn(PCZ)4]^2+^. It was observed that the fungicide propineb inhibited photosynthetic electron transport and disrupted photosystem II reaction center in *Nicotiana tabacum* [66]. The decrease in photosynthetic pigments (chlorophylls) may cause a decline in reducing sugars. Furthermore, 13.13% decrease in the ascorbic acid content in imidacloprid-treated potato samples as compared to the untreated ones [77]. In the same vein, a 20% reduction in ascorbic acid content and a 4% decrease in glucose content were observed in the treated tomato samples following the application of profenofos [78]. A study by Kengar et al. [79] on triazophos at lower concentrations demonstrated an upsurge in the reducing sugar content in spinach. An increased level of reducing sugar may be due to its non-conversion to non-reducing sugar. This might be a mechanism adopted by the plant to reduce the effect of triazophos stress. Shalaby [80] found that residues of thiamethoxam and chlorpyrifos significantly reduced the levels of total soluble sugars (%), glucose (mg/100 g), acidity (%), total soluble solids (%), ascorbic acid (mg/100 g), protein content (%), β-carotene (%), protein (%), and dry matter (%) in fresh treated okra fruits. Likewise, Shalaby [28] observed a reduction in the mean levels of total soluble solids, ascorbic acid, β-carotene, and acidity in tomatoes after 6, 9, and 15 days following profenofos spraying. Pirimiphos-methyl residues were found to have significant negative effects on the total sugar and ascorbic acid content in both tomato fruits and broad bean seeds [81]. In the same manner, acetamiprid and lambda-cyhalothrin significantly reduced the total sugar and ascorbic acid content of tomato fruit [82].

## 4. Conclusions

The study focused on evaluating the residues of PCZ and DFZ in tomatoes under various field conditions and processing methods, including washing with water, acetic acid, Na_2_CO_3_, and heating to produce tomato paste (the final product). It also assessed the impact of these treatments on macro- and micro-nutrient levels and tomato quality parameters via some scenarios. In the first scenario, the degradation of PCZ and DFZ ranged from 70% and 73% after six days of application to 99% and 98% after 15 days of spraying, respectively, with half-life (t_1_/_2_) values of 2.076 and 2.78 days. In the second scenario, the highest degradation of PCZ and DFZ was observed during the paste-making process, followed by Na_2_CO_3_, acetic acid, and water, when compared to the unwashed samples one day after application. In the third scenario, PCZ and DFZ treatments led to significant reductions in both internal macro- and micro-nutrient levels, as well as changes in quality parameters of tomato fruits, with a more pronounced effect observed following DFZ treatment. Our findings clearly demonstrate the critical role of processing techniques in reducing pesticide residues in tomato fruits, thereby contributing to a lower potential health risk for consumers.

## Figures and Tables

**Table 1:** Linearity range, correlation coefficient (R_2_), LOQ, LOD, precision, accuracy, and matrix effect of PCZ and DFZ in tomato fruits.

	Acetonitrile		Tomato Fruits	
	PCZ	DFZ	PCZ	DFZ
Range (mg/L)	0.01–5	0.01–5	0.01–5	0.01–5
R^2^	0.9999	0.9996	0.9997	0.9977
Slope	195.14	168.65	209.78	142.73
Intra-day repeatability (RSDr, %)	-	-	≤4.94	≤3.22
LOQ (mg kg^−1^)	-	-	0.1	0.1
LOD (mg kg^−1^)	-	-	0.033	0.033
Matrix effect (%)	-	-	7.5	−15.3

PCZ: Penconazole, DFZ: Difenoconazole, LOQ: Limit of Quantification, LOD: Limit of Detection.

**Table 2:** Recovery % and relative standard deviation (RSD%) of PCZ and DFZ in tomato fruits (n = 5).

Fungicides	Spiked Level (mg‧kg^−1^)	Average (%)	RSD (%)
PCZ	0.1	98.44	1.60
	0.5	97.86	4.83
	1	105.08	2.47
DFZ	0.1	97.64	1.44
	0.5	98.66	1.66
	1	100.08	3.22

PCZ: Penconazole, DFZ: Difenoconazole.

**Table 3:** Residue levels and dissipation behavior of PCZ and DFZ in tomato fruits under field conditions.

Intervals (Days)	PCZ			DFZ		
	Residues (mg/kg)	Degradation (%)	Persistence (%)	Residues (mg/kg)	Degradation (%)	Persistence (%)
0	0.79 ± 0.12	0	100	2.85 ± 0.24	0	100
1	0.66 ± 0.14	16.45	83.55	1.79 ± 0.27	37.19	62.81
3	0.44 ± 0.05	44.3	55.7	0.91 ± 0.08	68.07	31.93
6	0.23 ± 0.02	70.88	29.12	0.76 ± 0.04	73.33	26.67
9	0.12 ± 0.10	84.81	15.19	0.33 ± 0.0.3	88.42	11.58
12	0.09 ± 0.07	88.6	11.4	0.14 ± 0.04	95.08	4.92
15	0.002 ± 0.002	99.74	0.26	0.05 ± 0.04	98.25	1.75
PHI	12 days			9 days		
t_1/2_	2.08 days			2.78 days		
k	0.334 days ^−1^			0.249 days^−1^		

PCZ: Penconazole, DFZ: Difenoconazole, MRL: Maximum residue limit, PHI: pre-harvest intervals, t_1/2:_ Half-life residue, k: rate of degradation.

**Table 4:** Removal of Residue levels of PCZ and DFZ in tomato fruits after one day of application.

Process		Unwashed	Water	Acetic Acid	Sodium Carbonate	Paste
Residues (mg/kg)	PCZ	0.66 ± 0.14	0.44 ± 0.03	0.38 ± 0.07	UND	0
	DFZ	1.79 ± 0.27	0.31 ± 0.06	0.41 ± 0.11	0.30 ± 0.02	0.09 ± 0.08
Degradation (%)	PCZ	16.45	32.82	42.92	100	100
	DFZ	37.19	82.68	77.28	83.42	94.97
Processing Factor	PCZ	-	0.67	0.58	0	0
	DFZ	-	0.17	0.23	0.17	0

UND: undetectable; PCZ: penconazole; DFZ: difenoconazole.

**Table 5:** Health risk assessment of PCZ and DFZ on treated tomato fruits.

Parameters		Days						
		Initial	1	3	6	9	12	15
EADI	PCZ	0.1165	0.0973	0.0649	0.0339	0.0177	0.0132	0.0002
	DFZ	0.4203	0.264025	0.1342	0.1121	0.0486	0.0206	0.0073
HRI	PCZ	3.8841	3.245	2.1633	1.308	0.590	0.4425	0.0098
	DFZ	42.037	26.402	13.422	11.21	4.86	2.065	0.7375
Health risk	PCZ	Yes	Yes	Yes	Yes	No	No	No
	DFZ	Yes	Yes	Yes	Yes	Yes	Yes	No

PCZ: Penconazole, DFZ: Difenoconazole, Initial: One hour after spraying, EADI: Estimated Average daily intake, HRI: Health risk Indices, for difenoconazole was 0.01 mg/kg.

## Data Availability

The original contributions of this study are included in the article. Further inquiries can be directed to the corresponding authors.
